# Improving the antimicrobial efficacy against resistant *Staphylococcus aureus* by a combined use of conjugated oligoelectrolytes

**DOI:** 10.1371/journal.pone.0224816

**Published:** 2019-11-15

**Authors:** Elias L. Bazan, Lin Ruan, Cheng Zhou

**Affiliations:** 1 School of Chemical and Biomedical Engineering, Nanyang Technological University, Singapore; 2 Singapore Centre on Environmental Life Sciences Engineering (SCELSE), Nanyang Technological University, Singapore; Academia Sinica, TAIWAN

## Abstract

Two membrane-intercalating conjugated oligoelectrolytes (COEs), namely **COE-D8** and **COE-S6**, were combined to achieve enhanced antimicrobial efficacy. **COE-D8** has a shorter molecular length than **COE-S6** and is typical of effective antimicrobial COE molecules, presumably due to its prominent membrane disrupting function. In contrast, **COE-D6** exhibits lower efficacy against bacteria and lower toxicity toward mammalian cells. Surprisingly, after supplementing 8 μM **COE-S6**, the minimum inhibitory concentration (MIC) of **COE-D8** against methicillin-resistant *Staphylococcus aureus* (MRSA) was improved 8-fold, from 0.5 μM to 0.063 μM (0.050 μg mL^−1^). No increased toxicity toward mammalian cells was observed by the combination of COEs, as indicated by cytotoxicity measurements using the 3T3 cell line. Indeed, there is an extended ratio between the half maximal inhibitory concentration based on 3T3 cells to MIC against MRSA from 12 to greater than 256. Biophysical experiments using liposome models suggest that **COE-S6** promotes the interactions between **COE-D8** and lipid bilayers, which is in agreement with damages of cellular permeability and morphology, as observed by confocal microscopy and scanning electron microscopy. The application of a combined mixture of COEs further demonstrates their promising potential as a new class of antimicrobial agents with high efficacy and selectivity.

## Introduction

Resistance against conventional antibiotics poses a severe global threat that could negatively impact health and impose a substantial economic burden.[1–4] *Staphylococcus aureus* is a relevant example, since it rapidly acquired resistance toward sulfonamides and then evolved resistant strains to penicillin in the 1950s by increasing the production of β-lactamase.[5–8] Not long after penicillinase-stable methicillin was developed in 1960, methicillin-resistant *S*. *aureus* (MRSA) appeared in the following year by acquisition of the genetic element of SCC*mec* (staphylococcal cassette chromosome *mec*).[9, 10] MRSA has therefore acquired resistance to most β-lactam antibiotics, including more recently developed oxacillin, flucloxacillin and dicloxacillin.[11] Evolving resistance of *S*. *aureus* to other antibiotics, like tetracycline, linezolid, and fusidic acid, has also been reported.[12–14] New classes of antimicrobial agents effective against MRSA, and strategies for their use, are therefore urgently needed.[15–17]

Antimicrobial peptides (AMPs), also referred to as “host defense peptides”, provide a relevant point of discussion.[18–23] AMPs are thought to kill bacteria by destabilizing their membranes or cell walls through utilization of multiple complementary actions that help mitigate resistance development.[24, 25] Preparation costs and non-ideal chemical stability of AMPs limit large-scale clinical applications.[26] Antimicrobial cationic polymers that mimic AMPs are therefore under development with the goal of overcoming these limitations.[27–32] The molecular weight and chain sequence have been determined to be critical factors that influence the antimicrobial activities of these polymers.[33, 34] These factors, coupled with the dispersity of chain lengths and randomness of monomer arrangements in the polymer backbone, have the potential to cause batch-to-batch variations.[35–37]

**Fig 1:**
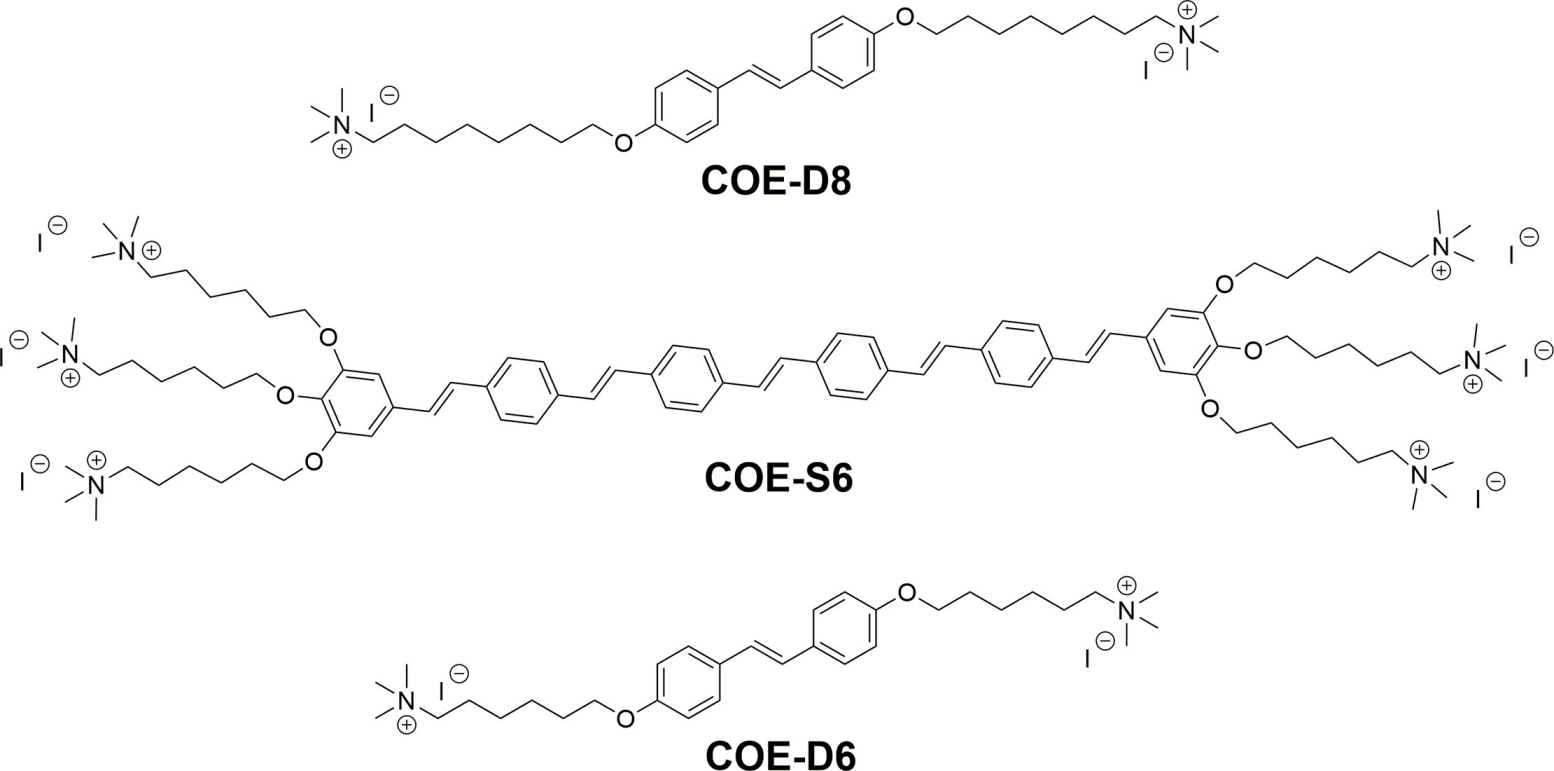
Chemical structures of COE-D8, COE-S6 and COE-D6.

Conjugated oligoelectrolytes (COEs) are a new class of antibiotics whose mechanism of action remains poorly understood and whose potential for widespread implementation requires further development.[38–42] COEs that exhibit antimicrobial activity typically contain a hydrophobic and linear conjugated backbone (such as oligo-phenylenevinylene) and terminal ionic groups. Their overall molecular structures are similar to the general distribution of charged and hydrophobic domains in lipid bilayers. As such, COEs can spontaneously intercalate and partition into cellular membranes.[43] The length of a COE regulates its impacts on lipid bilayers post intercalation.[44] As shown in Fig 1, **COE-D8** and **COE-D6** are typical and potent antimicrobial molecules, due to membrane disruption likely caused by a dimensional mismatch between the COE length and the lipid bilayers thickness.[40] In contrast, intercalation of the longer **COE-S6** exhibits negligible efficacy against bacteria. The elongated hydrophobic backbone of **COE-S6** indeed appears to act as a hydrophobic stabilizer for glycerophospholipid acyl chains and can even stabilize microbial membranes against environmental perturbations, for example, the presence of butanol in the medium.[45] In this work, we show the surprising effect of combining a “disruptor” **COE-D8** with the “stabilizer” **COE-S6** to achieve more effective antimicrobial activity as a prelude to advanced drug development steps.

## Materials and methods

### Materials, strains and instruments

The three COEs used in this work (**COE-D8**, **COE-S6** and **COE-D6**) were synthesized according to previous literature precedent.[40, 45] 1-Palmitoyl-2-oleoyl-phosphatidylethanolamine (POPE) and 1-palmitoyl-2-oleoyl-phosphatidylglycerol (POPG) were purchased as chloroform stock solutions from Avanti Polar Lipids. The Propidium Iodide and SYTO 9 fluorescent dyes were purchased from Invitrogen as solid stocks. Four *S*. *aureus* strains were used in this study, including two susceptible strains (*S*. *aureus* 25923 and *S*. *aureus* 29213) and two resistant strains (MRSA BAA-40 and ORSoA <oxacillin-resistant *S*. *aureus*>). MHB (Mueller Hinton Broth, BD BLL) media and (1×) PBS (Phosphate buffered saline, pH = 7.2) were prepared and autoclaved before use. The 3T3 cell line was used as a model mammalian cell in the *in vitro* cytotoxicity measurements. Fluorescent micrographs were captured using a Leica SP8 confocal microscope. Scanning electron microscopy (SEM) was performed using an FESEM (JEOL JSM-6700F) instrument for cellular morphology characterizations. Differential scanning calorimetry (DSC) curves of vesicle solutions were measured using a Nano DSC instrument (TA Instruments) at heating and cooling rates of 1°C min^−1^.

### Determination of minimum inhibitory concentration

The minimum inhibitory concentrations (MICs) of relevant COEs were determined using a broth microdilution method in a 96-well plate. Briefly, COEs were diluted via a 2-fold dilution series in 100 μL of MHB to achieve twice the concentration needed in the MIC determination. The chequerboard broth microdilution assay used to determine the synergistic antimicrobial activities operates similarly, except that the 100 μL MHB media in each well contains two COEs. Subsequently, each well was inoculated with 100 μL of 1 × 10^6^ colony forming units (CFU) mL^−1^ of *S*. *aureus* in MHB to achieve an inoculum density of 5 × 10^5^ CFU mL^−1^. The plates were then incubated at 37°C with shaking (200 rpm) for 18 hours. MIC values were determined as the lowest COE concentration at which microbial growth was completely inhibited by comparing the final optical density at 600 nm (OD_600_) with positive and negative growth controls using a plate reader (TECAN Infinite M200). All results were repeated in triplicate.

### Determination of fractional inhibitory concentration index

Fractional inhibitory concentration (FIC) indices, based on the chequerboard broth microdilution assay, were calculated according to the following equation:

$$FIC=FIC_{A}+FIC_{B}=\frac{C_{A}}{MIC_{A}}+\frac{C_{B}}{MIC_{B}}$$

where FIC_A_ is the FIC contributed from compound A; FIC_B_ is the FIC contributed from compound B, MIC_A_ is the MIC of compound A alone; MIC_B_ is the MIC of compound B alone; C_A_ and C_B_ are the concentrations of compound A and compound B, respectively, when they exhibit bacterial growth inhibition after combined use. Synergy is defined when FIC ≤ 0.5, while antagonism is defined when FIC ≥ 4.[46]

### Bacteria killing assay

A stationary-phase culture of MRSA BAA-40, incubated in MHB at 37°C with shaking (200 rpm) for 36 hours, was harvested by centrifuge (6000 rpm, 5 mins). After removing the supernatant, the cells were washed twice with PBS solution, and were then diluted to approximately 5 x 10^7^ CFU mL^−1^ in PBS by measuring the OD_600_ of the suspension. The bacteria suspension was subsequently mixed with equivalent volumes of **COE-D8** or **COE-S6** solution in PBS. The tube, under corresponding COE treatments, was incubated at room temperature with shaking (150 rpm). After 6 hours, the bacteria suspensions were serially diluted 10-fold in PBS and plated on MHB agar plates to enumerate cell number (CFU mL^−1^).

### Cytotoxicity measurements

The *in vitro* biocompatibility investigation for COEs was carried out using MTT assays based on the 3T3 cell line and HepG2 cell line (purchased from ATCC^®^). The mammalian cells were cultured in a 96-well plate at 37°C from an initial inoculum of 10,000 cells in each well containing 200 μL DMEM (Dulbecco's Modified Eagle Medium). For the HepG2 cells, 1% of MEM NEAA (Non-Essential Amino Acids, Gibco^™^) was added as the supplement in the medium. After 24 hours incubation, all the media were removed, and the cells were washed with PBS. Gradually diluted COE solutions in DMEM were prepared according to specific requirements and then 200 μL were added to the cell cultures. For control cells, pure DMEM was added as the control experiments. After incubation at 37°C for 24 hours, all solutions in the plate were removed and the cells were washed with PBS, before the addition of 200 μL of 1 mg mL^−1^ MTT (3-(4,5-dimethylthiazol-2-yl)-2,5-diphenyltetrazolium bromide) solution in DMEM. After incubation at 37°C for another 4 hours, all solutions in the plate were removed and 200 μL of DMSO (dimethyl sulfoxide) were added and mixed in each well. The cell viabilities were determined based on the absorbance at 570 nm relative to the control wells. The results were repeated in five replicates.

### Hemolysis assay

Fresh human blood was collected from a healthy donor (age 30, Male). 1 mL blood was mixed with 9mL PBS and centrifuged at 1,000 rpm for 5 min. Red blood cell pellets were collected and subsequently washed with PBS three times and diluted to a final concentration of 5% v/v. The COE was dissolved in PBS and two folds serial diluted in a 96-well microplate (Nunc^TM^, ThermoScientific). 50 μL red blood cell stock was mixed with 50 μL COE solution in each well and incubated for 1 h at 37°C under shaking. The microplate was centrifuged at 1,000 rpm for 10 min. 80 μL aliquots of the supernatant were then transferred to a new 96-well microplate and diluted with another 80 μL of PBS. Hemolytic activity was calculated by measuring absorbance at 540 nm using a 96-well plate spectrophotometer (Benchmark Plus, BIO-RAD). Triton X-100 (0.1% in PBS) which is able to lyse red blood cells completely was used as positive control, while PBS was used as negative control. The hemolysis percentage was calculated using the following formula:

$$Hemolysis\left(\%\right)=\frac{O_{c}-O_{b}}{O_{t}-O_{b}}\times 100\%$$

where O_c_ is the absorbance of COE-treated sample, O_b_ is the absorbance of negative control and O_t_ is the absorbance of positive control. The results were repeated in four replicates.

### Confocal microscopy

Stationary-phase MRSA cells were harvested by centrifuge (6000 rpm, 5 mins), washed twice with PBS solution, and resuspended in PBS to a density of OD_600_ = 1.0 before use. 500 μL MRSA solutions were mixed with equal volume COE solutions in PBS to achieve four different conditions (non-treated, 16 μM **COE-S6** treated, 8 μM **COE-D8** treated, both 8 μM **COE-D8** and 16 μM **COE-S6** treated). After treatment with COEs for 2 hours at room temperature with shaking (150 rpm), cells were harvested by centrifuge (6000 rpm, 5 mins), and then resuspended in PBS to a cell density with OD_600_ = 1.0. Then, 200 μL of MRSA solutions were mixed with an equal volume PBS solution that contains propidium iodide and SYTO 9 fluorescent dyes. The final cell density is OD_600_ = 0.5 (~3 × 10^8^ CFU mL^−1^), and the concentration of each dye will be 3 μM for SYTO 9 stain and 15 μM for propidium iodide. The stained cells were incubated at room temperature in the dark for 15 minutes before sample preparation. The red propidium iodide fluorescent channel was observed by excitation at 570 nm and the emission was collected in the range of 600 − 630 nm. The green SYTO 9 fluorescent channel was observed by excitation at 488 nm and the emission was collected in the range of 500 − 530 nm.

### SEM sample preparation

Stationary-phase MRSA cells were harvested by centrifuge (6000 rpm, 5 mins), washed twice with PBS solution, and then resuspended in PBS to an adjusted density of OD_600_ = 1.0. 200 μL MRSA solutions were mixed with equivoluminal corresponding COE solutions in PBS to achieve four different conditions (non-treated, 8 μM **COE-S6** treated, 16 μM **COE-D8** treated, both 8 μM **COE-S6** and 16 μM **COE-D8** treated). After removing the PBS solution by centrifuge, the microbes were immediately fixed with 1 mL of 2.5% glutaraldehyde aqueous solution and kept at 4°C overnight. Next, 10 μL of bacteria suspension was spotted on to the silicon wafer. After drying in air, the bacteria were dehydrated with a series of graded ethanol aqueous solutions (20% − 100%). After drying overnight, the samples were coated with platinum before SEM imaging.

### Small multilamellar vesicles preparation

Chloroform solutions of POPE and POPG were mixed to a molar ratio of 85:15 and dried under a gentle stream of nitrogen. The dried lipid was further desiccated overnight to obtain a thin lipid film. Rehydration of the dried film was carried out by adding Tris-NaCl buffer (25 mM Tris, 150 mM NaCl, pH = 7.5), to a lipid concentration of 5 mg mL^−1^, followed by incubation at 35°C for 2 hours under constant stirring using a magnetic stirrer at 300 rpm. The resulting vesicles were extruded using a 100 nm membrane at 45°C 21 times. The resulting small multilamellar vesicles (SUVs) were kept at 4°C until further use. A vesicle suspension was diluted to 1 mg mL^−1^ in Tris-NaCl buffer under different concentrations **COE-D8** treatment with/without 8 μM **COE-S6** supplement before carrying out the DSC measurements.

## Results and discussion

### Synergistic antimicrobial activity

**Fig 2:**
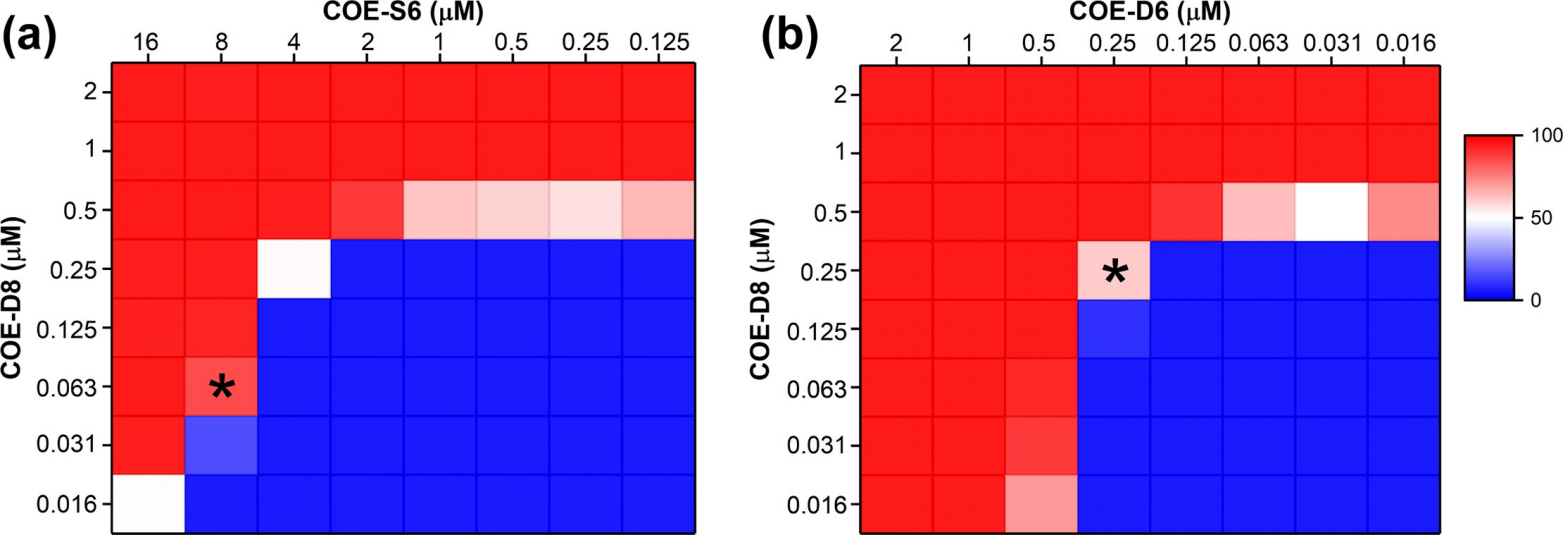
Growth inhibition percentage of MRSA in MHB at 37°C for 18 hours after treatment with different concentrations **COE-D8** with synergistic use of (a) **COE-S6** or (b) **COE-D6**. The bacterial growth inhibition was determined based on the final optical density at 600 nm (OD_600_) after 18 hours incubation, with positive and negative growth controls as 0% (represented in blue color) and 100% (represented in red color) inhibition, respectively. The low percentage value or more blue color represents higher cell density. The FIC values in the wells as marked by asterisk are 0.16 and 1 for **COE-S6** (left) and **COE-D6** (right) synergy experiment sets, respectively.

MIC values of COEs against *S*. *aureus* strains were measured via a broth microdilution method using the procedure described in the preceding Materials and methods section. As previously mentioned, the length of the conjugated phenylenevinylene component modulates the *in vitro* antimicrobial activity of the corresponding COE. COEs with a molecular length shorter than the thickness of lipid bilayers (≈ 4nm) are typically inhibitory.[40] Consistent with these previous reports, **COE-D8** exhibits potent antimicrobial activity, with an MIC value of 0.5 μM against the reference resistant strain MRSA BAA-40. For comparison, the MIC value obtained for the longer **COE-S6** against MRSA (256 μM) is more than 500 times larger under the same experimental conditions. Their synergistic antimicrobial activities were subsequently investigated by associative use of the two COEs in a chequerboard broth microdilution assay (Fig 2A), where the concentration of **COE-D8** ranges from 2 to 0.016 μM vertically and the concentration of **COE-S6** ranges from 16 to 0.125 μM horizontally. The bacterial growth inhibition was determined based on the final OD_600_ values after 18 hours incubation and were compared to the positive (MHB contains only bacteria) and negative (pure MHB) growth controls. When the concentration is below 2 μM, **COE-S6** exhibits less influence on the MRSA growth inhibition than that posed by **COE-D8**. A sharply reduced inhibiting concentration of **COE-D8** down to a remarkable 0.063 μM was seen when 8 μM **COE-S6** was applied. The FIC index for this well (marked by asterisk in Fig 2A) was calculated to be 0.16. As the **COE-S6** further increases to 16 μM, a lower FIC value of 0.12 was achieved as the **COE-D8** concentration is reduced to 0.031 μM. These FIC values are below 0.5, indicating that the combination of **COE-D8** and **COE-S6** exhibits a significant antimicrobial synergy effect.

The COE **COE-D6** (Fig 2B) was also employed in synergy experiments with **COE-D8**. **COE-D6** is another short, membrane-disrupting antimicrobial COE with an effective MIC value of 0.5 μM against MRSA. However, in the chequerboard broth microdilution assay, the lowest FIC value calculated among the cell growth inhibited wells is 1 (> 0.5), where the concentrations for **COE-D8** and **COE-D6** are half of their MICs respectively (marked by asterisk in Fig 2B). This result indicates that there is no synergistic effect for the **COE-D8** and **COE-D6** combination, possibly due to their similar impacts on the membrane.

### General applicability studies

**Fig 3:**
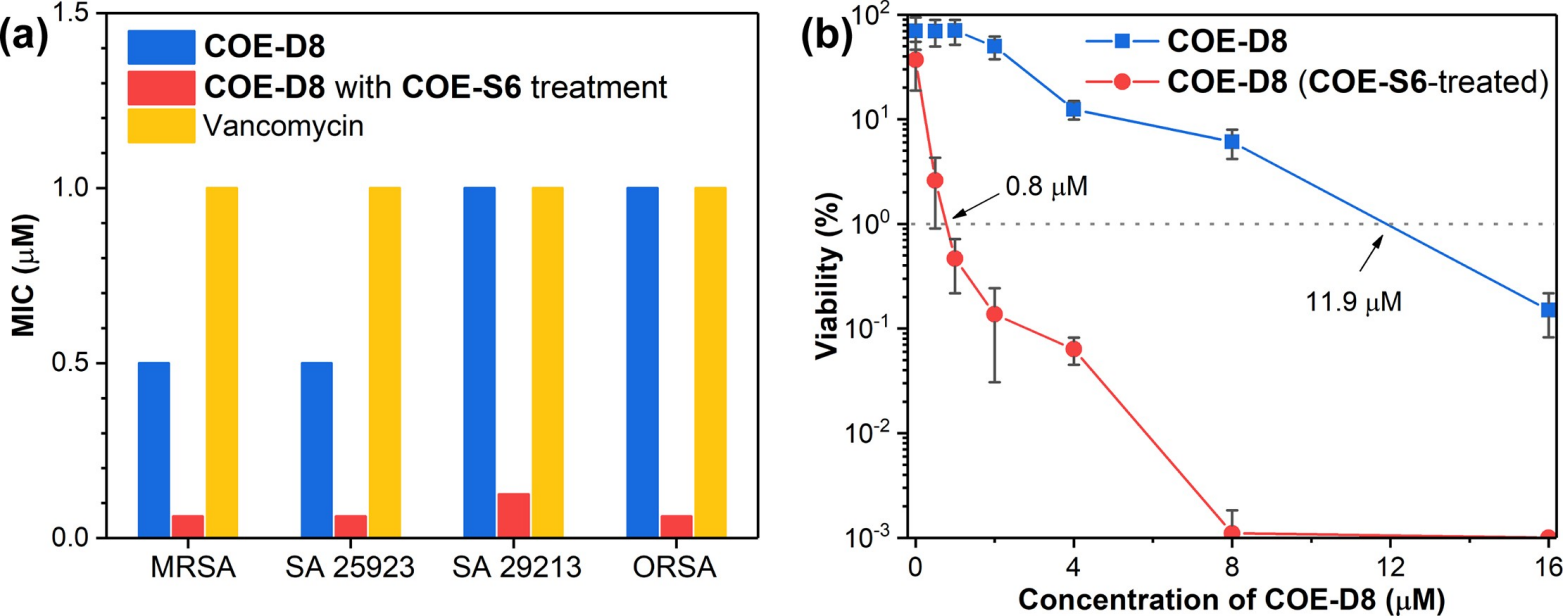
(a) MIC of **COE-D8** against four *S*. *aureus* strains as tested in MHB at 37°C for 18 hours, and (b) cellular viability of MRSA after treatment with different concentrations **COE-D8** in PBS solution with or without a supplement of 8 μM **COE-S6** for 6 hours.

According to the results in the synergistic experiments, 8 μM of **COE-S6** was selected as the supplement material to conduct further MIC measurements for **COE-D8**. The goal of these studies is to explore the general applicability of the **COE-S6** sensitization. Besides MRSA, three more *S*. *aureus* strains were employed, including two susceptible strains (*S*. *aureus* 25923 and *S*. *aureus* 29213) and one resistant strain ORSA (oxacillin-resistant *S*. *aureus*). As shown in Fig 3A, the MIC value of **COE-D8** against both MRSA and *S*. *aureus* 25923 decreases from 0.5 to 0.063 μM with the 8 μM **COE-S6** supplement. An MIC of 1 μM is needed for **COE-D8** alone to inhibit growth of *S*. *aureus* 29213 and ORSA, however the 8 μM **COE-S6** treatments shift their MIC values to 0.125 and 0.063 μM, respectively. The antimicrobial activity provided by the COE synergetic method is potent, especially when compared to the “last resort” vancomycin, which exhibit a MIC value of 1 μM against MRSA under the same conditions (Fig 3A). The COE combination method was applied toward two Gram-negative *Escherichia coli* strains (K12 and UTI89). However, the MIC of **COE-D8** against *E*. *coli* cells remains unchanged (8 μM) after 8 μM **COE-S6** supplement in MHB. Thus, the effect appears to be specific to the type of cell; the underlying reasons for selectivity are likely related to differences in interaction with different cell wall and/or membrane characteristics, the details of which would require additional mechanistic studies.

The potent antimicrobial activities provided by this COE synergistic method were further confirmed using bacterial killing assays, which measure the number of viable cells with or without **COE-S6** supplement after different concentrations of **COE-D8** treatments (from 0 to 16 μM). As shown in Fig 3B, when the applied **COE-D8** concentration is 1 μM, ~70% of MRSA cells related to the initial incubation are still viable after 6 hours of treatment at room temperature in PBS, but the viability percentage decreases to ~0.5% in the presence of 8 μM **COE-S6**. According to the concentration-dependence bacteria-kill curves, it is estimated that approximately 11.9 μM **COE-D8** is needed to eliminate 99% MRSA viability, while the **COE-S6** supplement reduces **COE-D8** demand to 0.8 μM under the same conditions. These results demonstrated that the bactericidal activities of **COE-D8** against *S*. *aureus* can also be enhanced by its **COE-S6** partner.

### Cytotoxicity measurements

**Fig 4:**
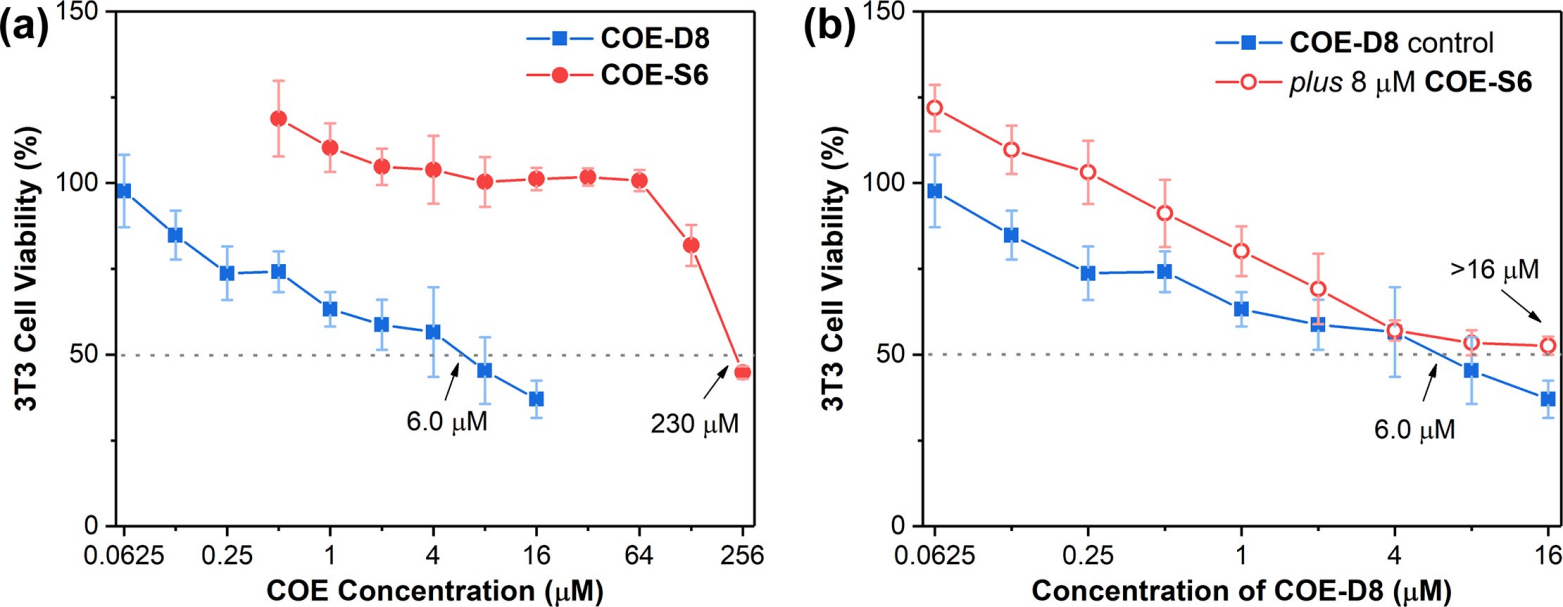
Cytotoxicity measurements based on the 3T3 cell line after treatment with corresponding COEs for 24 hours. Cellular viabilities after treatment with (a) different concentrations of **COE-D8** or **COE-S6**, and (b) the different concentrations of **COE-D8** with or without 8 μM **COE-S6** supplement.

Biocompatibility with mammalian cells is also a critical factor for an antibiotic agent in addition to its antimicrobial performance, especially in synergetic experiments where the additional compound may cause supererogatory toxic effects. *In vitro* cytotoxicity measurements based on the 3T3 cell line were performed with the COEs (Fig 4). After a 24-hour treatment, a half maximal inhibitory concentration (IC_50_) of 230 μM was observed for **COE-S6**, suggesting negligible toxicity to mammalian cells, especially when the applied concentration is below 64 μM (Fig 4A). Thus, **COE-S6** provides a wide concentration window for use as supplementary agent without bring additional toxicity. The corresponding IC_50_ values for **COE-D8** is 6.0 μM.

Changes in cytotoxicity caused by the combined use of **COE-D8** with **COE-S6** was subsequently investigated (Fig 4B). No additional toxicity was caused by 8 μM **COE-S6** supplement, and the IC_50_ of **COE-D8** increased to more than 16 μM as the cytotoxicity graph curved upward. Thus, the ratio between IC_50_ (based on the 3T3 cell line) to MIC (against MRSA) was extended by a factor greater than 20 (from 12 to more than 256). The results agree with the individual cytotoxicity experiments that 8 μM **COE-S6** alone shows almost no toxic effects on 3T3 cells with cell viability of ~100% (Fig 4A). It is noted that slightly exceeded 100% viability values were calculated for **COE-S6** treated 3T3 cells in Fig 4, indicating that the cell metabolic activities were probably promoted by the low concentration **COE-S6** supplement.[47] Furthermore, cytotoxicity measurements based on HepG2 cell line (S1 Fig) and hemolysis measurements based on human red blood cells (S2 Fig) were performed, and both experiments verified that the 8 μM **COE-S6** supplement does not bring additional toxicity to mammalian cells. It is interesting that the **COE-S6** supplement can selectively enhance the membrane disruption activity of **COE-D8** on *S*. *aureus* cells (Fig 2 and Fig 3). One possible explanation is the COE itself (including both **COE-D8** and **COE-S6**), decorated with cationic side chains, can preferentially bind to anionic bacterial membranes over zwitterionic mammalian cell membranes. Another possible explanation is that **COE-S6** may exhibit specialized affinity to *S*. *aureus* membranes as a result of its unique six-charge distributed in the terminals of elongated backbone. For example, as illustrated previously, the COE synergetic method works well on *S*. *aureus* but has no effect on bacteria *E*. *coli*. In short, these antimicrobial and cytotoxicity measurements suggest that the combined use of **COE-D8** and **COE-S6** can achieve high antimicrobial efficacy and extend the selectivity at the same time.

### Biophysical insights

**Fig 5:**
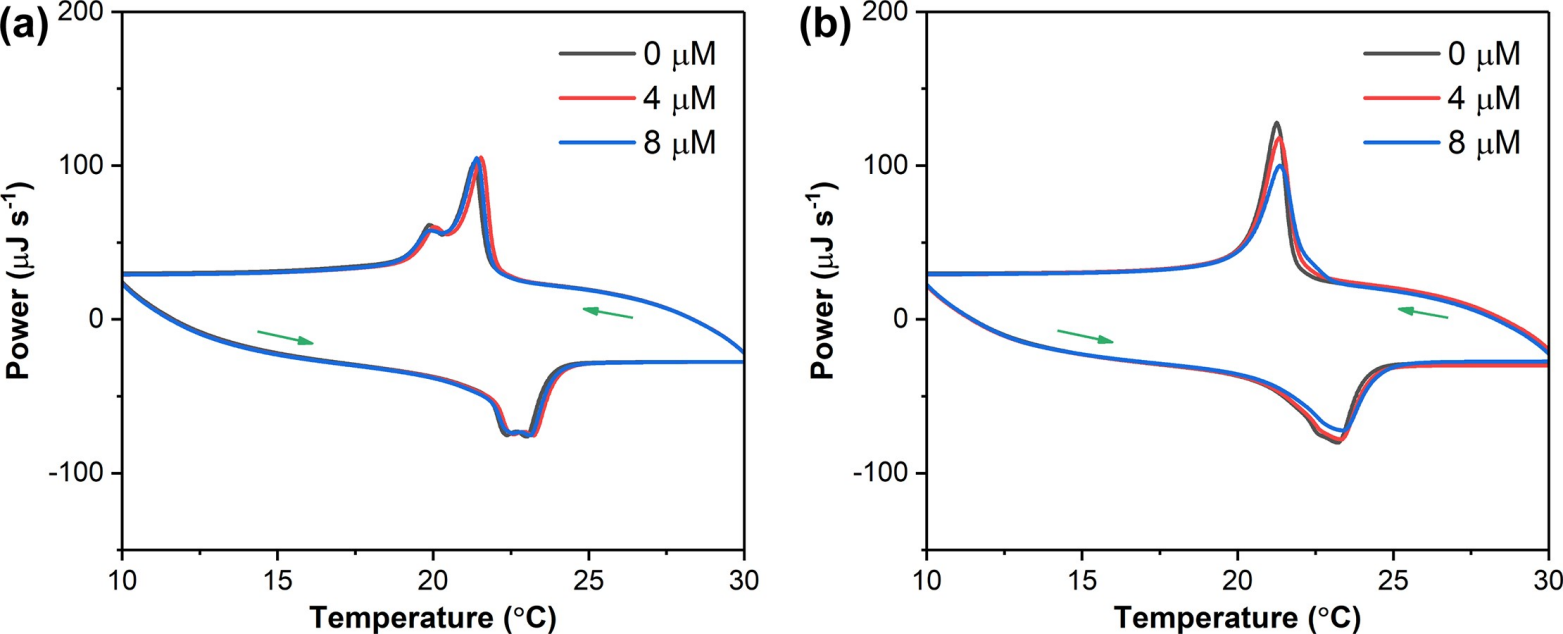
DSC curves at heating and cooling rates of 1°C min^−1^ of 1 mg mL^−1^ (a) neat POPE:POPG SUVs and (b) 8 μM **COE-S6**-treated SUVs after treatment with different concentrations **COE-D8**.

Having established the antimicrobial activity and cytotoxicity effect of combinations of **COE-D8** and **COE-S6**, we sought to understand the improved antimicrobial performance effect and to obtain information regarding membrane interactions. Small unilamellar vesicles (SUVs), composed of 1-palmitoyl-2-oleoylphosphatidylethanolamine (POPE) and 1-palmitoyl-2-oleoylphosphatidylglycerol (POPG) in a molar ratio of 85:15, were extruded through use of a 100 nm membrane and were used to model bacterial membranes in differential scanning calorimetry (DSC) experiments (Fig 5). The main gel-to-liquid-crystal transition peaks during heating and cooling processes represent the cooperative conformational rearrangement of aliphatic chains.[48] A concentration range of **COE-D8** from 0 to 8 μM was applied to 1 mg mL^−1^ vesicles, and no obvious change in intensity or shape was seen for the DSC peaks, suggesting that **COE-D8** alone, in this concentration range, does not impact SUV thermal transitions.[40] Under the same concentration condition, the antibiotic vancomycin also exhibits no effect on SUV thermal transitions (S4A Fig), even though the applied concentration was increased to 32 μM (S4B Fig). In contrast, the high concentration of **COE-D8** (32 or 64 μM) leads to significant damages the SUV structures (S4C Fig).

**COE-S6**-treated SUVs, made by adding 8 μM **COE-S6** to 1 mg mL^−1^ SUVs in Tris-NaCl buffer, show a significant change in the DSC traces relative to the initial curve (Fig 5B). Specifically, one observes that the shoulder peak disappeared during the cooling process, which is most likely a result of membrane intercalation by **COE-S6**. Under the presence of **COE-S6**, a gradually broadened DSC peak with decreased intensity is observed as **COE-D8** increases from 0 to 8 μM, demonstrating that the low concentration **COE-D8** has caused enhanced perturbations in the hydrophobic domains of lipids. Overall, these biophysical experiments indicate that **COE-S6** containing SUVs are more sensitive toward the presence of **COE-D8**. It is worth pointing out that the details of how COEs interact with lipid bilayers remain under investigation. The findings that **COE-S6** can promote the interactions between **COE-D8** and lipid bilayers indicate a similar process may occur on microbes, thus enhancing the antimicrobial performance after their combined use.

### Confocal microscopy

Membrane integration after COE treatment was examined using confocal fluorescent microscopy by using bacterial viability staining with two fluorescent nucleic acid stains, SYTO 9 and propidium iodide (PI). SYTO 9 can enter both live and dead bacterial cells and stain them so that they exhibit green fluorescence after nucleic acid binding. In contrast, PI can only penetrate compromised membranes, which may be attributed to dead or dying cells. Hence, when both dyes are present, the intact membrane cells will exhibit green emission from SYTO 9, while the disrupted membrane cells will show red PI fluorescence due to the stronger affinity of PI which can displace SYTO 9 from nucleic acids.[49, 50]

**Fig 6:**
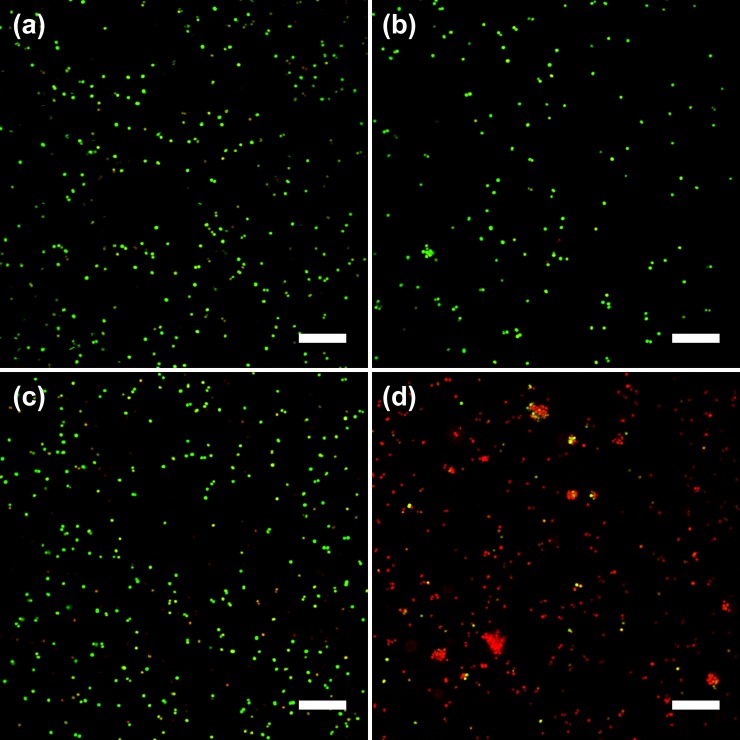
Merged fluorescence micrographs of MRSA cells stained by propidium iodide/SYTO 9 after different COE treatments in PBS for 2 hours. (a) Non-treated, (b) 8 μM **COE-S6** treated, (c) 8 μM **COE-D8** treated, (d) both 8 μM **COE-D8** and 8 μM **COE-S6** treated. Scale bars are 20 μm. The propidium iodide fluorescent channel (presented in red color) was observed by excitation at 570 nm and the emission was collected in the range of 600 − 630 nm. The SYTO 9 fluorescent channel (presented in green color) was observed by excitation at 488 nm and the emission was collected in the range of 500 − 530 nm.

MRSA cells were treated with corresponding COEs for 2 hours before staining with SYTO 9 and PI. Due to the higher density of cells needed for observation by microscopy (OD_600_ = 0.5) relative to MIC measurements, a higher **COE-D8** concentration of 8 μM was applied during the treatment. The brightfield and brightfield-fluorescence merged micrographs of MRSA were shown in S3 Fig. As shown in Fig 6A, nearly all control MRSA cells (without COE treatment) exhibit green fluorescence from SYTO 9, in agreement with their intact cellular membrane structures. A slightly increased but still low proportion of MRSA was penetrated by red PI after individually treating with 8 μM **COE-S6** (Fig 6B) or 8 μM **COE-D8** (Fig 6C). After applying both **COE-S6** (8 μM) and **COE-D8** (8 μM), the vast majority of MRSA cells contain PI, presumably as a result of damaged cell membrane/wall characteristics (Fig 6D).

### Scanning electron microscopy

**Fig 7:**
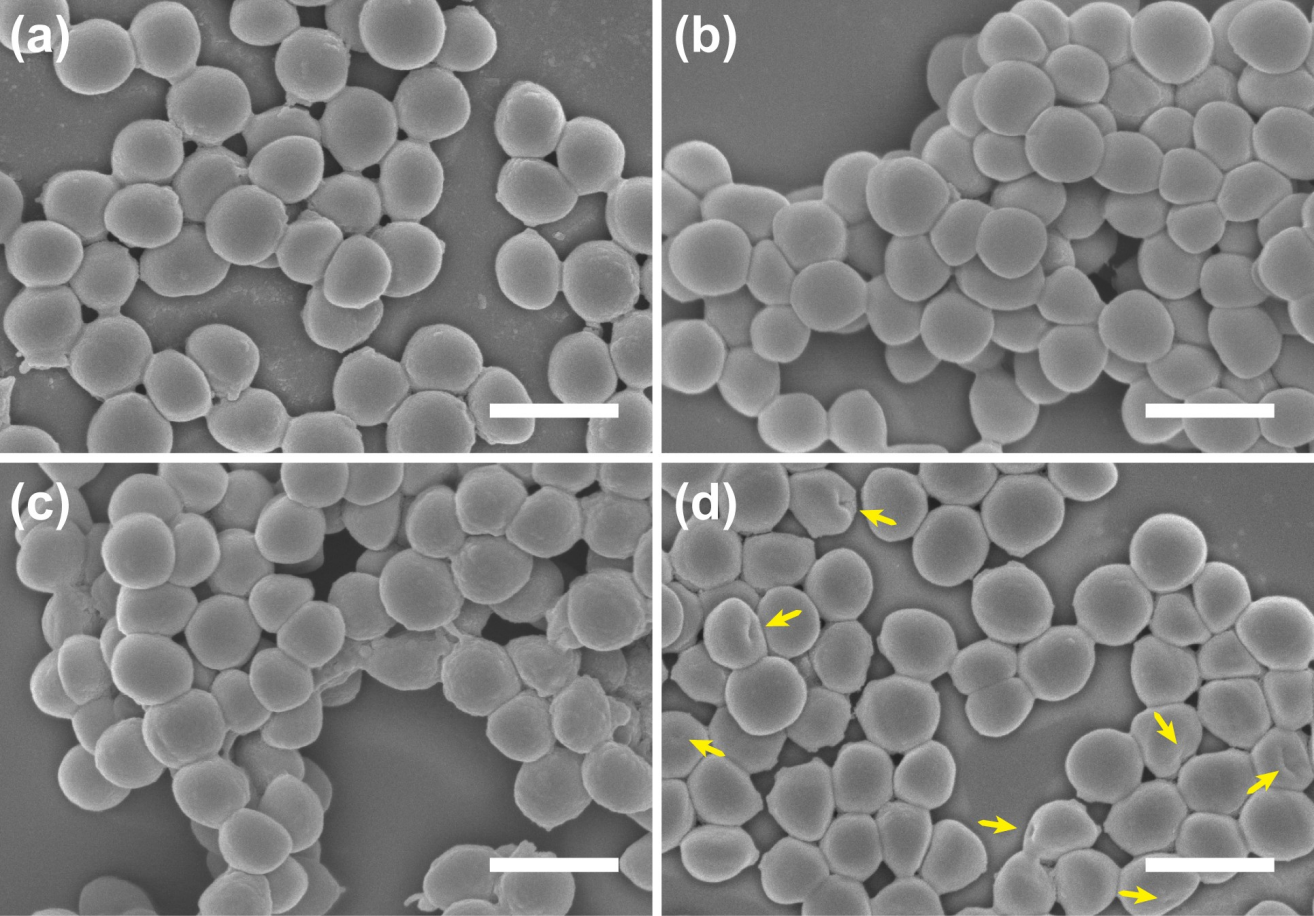
SEM images of MRSA after treatment with (a) no COE, (b) 8 μM **COE-S6** alone, (c) 16 μM **COE-D8** alone, (d) both 16 μM **COE-D8** and 8 μM **COE-S6**, for 4 hours in PBS solution. Scale bars correspond to 1 μm.

Cellular morphology post COE treatment was examined by using scanning electron microscopy (SEM). The results of these studies are shown in Fig 7. In these experiments, MRSA cells (OD_600_ = 0.5) in PBS buffer were treated with COEs under four conditions: non-treated, only **COE-S6** treated, only **COE-D8** treated, and the combined **COE-S6** and **COE-D8** treatment. Similar to the MRSA controls (without COE treatment, Fig 7A), characteristic coccus-shaped cells with smooth outer surfaces were observed for the group treated with individual 8 μM **COE-S6** or 16 μM **COE-D8** (Fig 7B and 7C). A natural antimicrobial peptide vancomycin was selected as a control in SEM measurements, and no obvious morphology change could be observed (S5A Fig), even though the applied vancomycin concentration was increased to 64 μM (S5B Fig). By contrast, the 64 μM **COE-D8** treatment collapses the MRSA envelope morphology. These results are in good agreement with the DSC measurements and suggest that the **COE-D8** employs the membrane disruption antimicrobial mechanism.

As shown in Fig 7D, after the synergetic implementation of 8 μM **COE-S6** and 16 μM **COE-D8** (Fig 7D), a greater abundance of pitting in the membranes can be observed. The surface features of both **COE-S6** and **COE-D8** treated MRSA cells, as surveyed by confocal microscopy and SEM, supports the biophysical experiment results that the existence of **COE-S6** promotes interactions between **COE-D8** and lipid bilayers, thus the increased membrane disruption confers a significantly enhanced antimicrobial performance for this synergetic COE strategy.

## Conclusions

Two membrane-intercalating COEs containing different backbone lengths were synergistically used against *S*. *aureus* strains. The short **COE-D8** is a potent antimicrobial molecule due to the effective membrane disruption likely caused by a dimensional mismatch between **COE-D8** and lipid bilayers. The longer **COE-S6**, for which intercalation leads to membrane stabilization, has a larger MIC value. After a supplement of 8 μM **COE-S6**, the antimicrobial activities of **COE-D8** were significantly promoted with a MIC value of 0.063 μM (0.050 μg mL^−1^) against both “superbugs” MRSA and ORSA. The results are worth considering relative to other membrane-disrupting antimicrobial agents, such as the “last resort”, vancomycin, which has an MIC against MRSA of 1 μM.[51] Moreover, no additional mammalian toxicity was induced by the **COE-S6** supplement as indicated by *in vitro* cytotoxicity measurements. Thus, an extended selectivity was seen as judged by the increase in the ratio between IC_50_ (based on 3T3 cells) to MIC (against MRSA) from 12 to more than 256. Biophysical experiments using liposome models provide evidence that the **COE-S6** enhances the membrane-disrupting effects of **COE-D8** on lipid bilayers, thus compromising membrane integrity. The high antimicrobial efficacy and low cytotoxicity, provided by this synergistic method, demonstrate the promising antimicrobial potential by the combined use of COEs.

## Supporting information

S1 Fig Cytotoxicity measurements based on the HepG2 cell line after treatment with corresponding COEs for 24 hours. Cellular viabilities after treatment with (a) different concentrations of **COE-D8** or **COE-S6**, and (b) the different concentrations of **COE-D8** with or without 8 μM **COE-S6** supplement.
(TIF)

S2 Fig Hemolysis measurements after treatment with corresponding COEs for 1 hours at 37°C. Hemolysis percentages after treatment with (a) different concentrations of **COE-D8** or **COE-S6**, and (b) the different concentrations of **COE-D8** with or without 8 μM **COE-S6** supplement.
(TIF)

S3 Fig Brightfield and brightfield-fluorescence merged micrographs of MRSA cells stained by propidium iodide/SYTO 9 after different COE treatments in PBS for 2 hours. (a, b) Non-treated, (c, d) 8 μM **COE-S6** treated, (e, f) 8 μM **COE-D8** treated, (g, h) both 8 μM **COE-D8** and 8 μM **COE-S6** treated. Scale bars are 20 μm. The propidium iodide fluorescent channel (presented in red color) was observed by excitation at 570 nm and the emission was collected in the range of 600 − 630 nm. The SYTO 9 fluorescent channel (presented in green color) was observed by excitation at 488 nm and the emission was collected in the range of 500 − 530 nm.
(TIF)

S4 Fig DSC curves at heating and cooling rates of 1°C min^−1^ of 1 mg mL^−1^ POPE:POPG SUVs after different treatments. (a) Different concentration of vancomycin, (b) without or with 32 μM **COE-D8** or vancomycin, (c) without or with 64 μM **COE-D8**.
(TIF)

S5 Fig SEM images of MRSA after different treatments for 4 hours in PBS solution. (a) Control, (b) 16 μM vancomycin, (c) 64 μM vancomycin and (d) 64 μM **COE-D8**. Scale bars correspond to 1 μm.
(TIF)

S1 Tables Raw data in this work. (XLSX)
