# Supplementary figures and images for: Improving the antimicrobial efficacy against resistant Staphylococcus aureus by a combined use of conjugated oligoelectrolytes
Source: PLoS One. 2019 Nov 15;14(11):e0224816. doi: 10.1371/journal.pone.0224816 (PMC6857938; doi:10.1371/journal.pone.0224816)

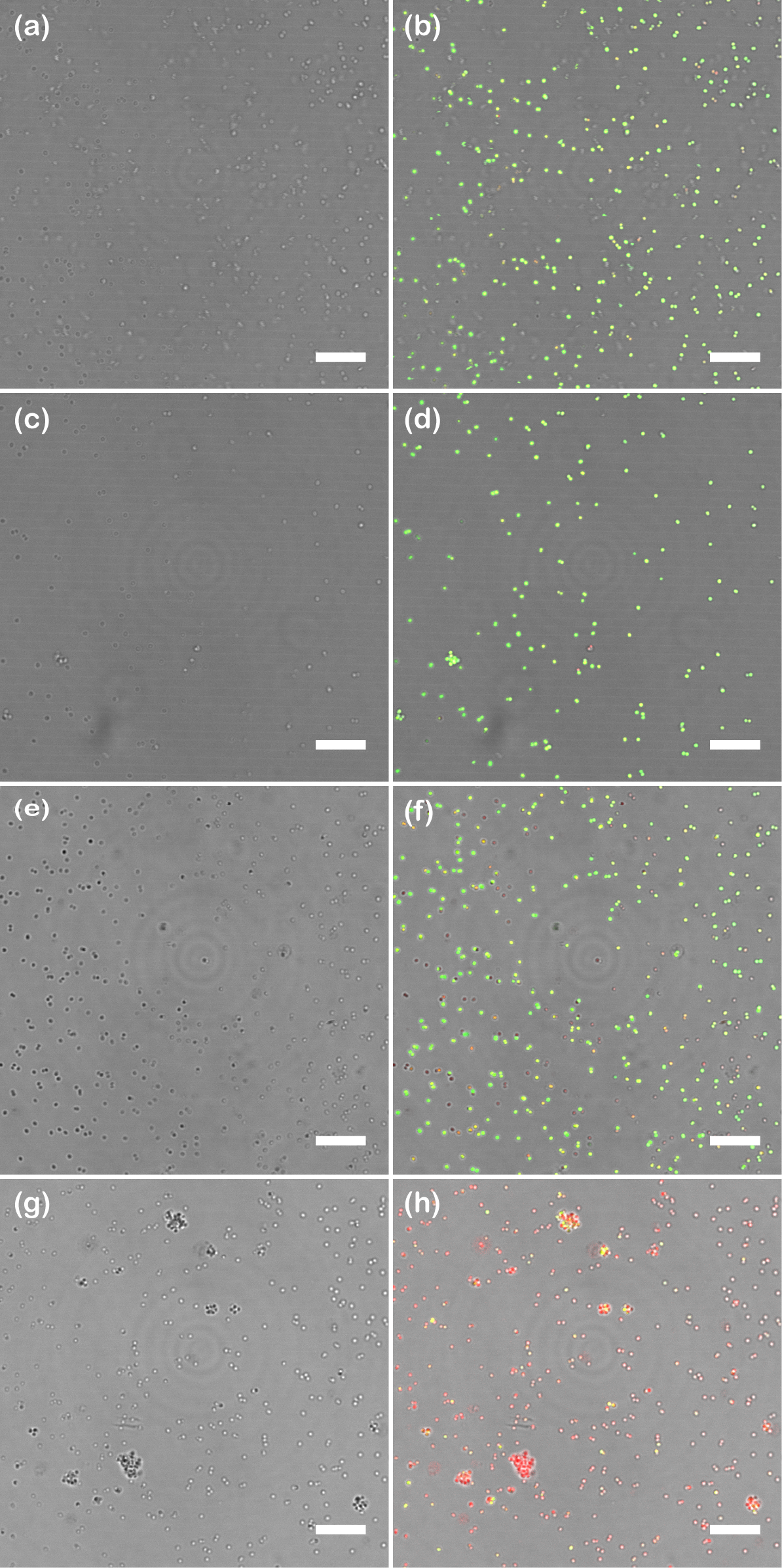

Supplement: S3 Fig — (a, b) Non-treated, (c, d) 8 μM COE-S6 treated, (e, f) 8 μM COE-D8 treated, (g, h) both 8 μM COE-D8 and 8 μM COE-S6 treated. Scale bars are 20 μm. The propidium iodide fluorescent channel (presented in red color) was observed by excitation at 570 nm and the emission was collected in the range of 600 − 630 nm. The SYTO 9 fluorescent channel (presented in green color) was observed by excitation at 488 nm and the emission was collected in the range of 500 − 530 nm. (TIF) [file pone.0224816.s003.tif]

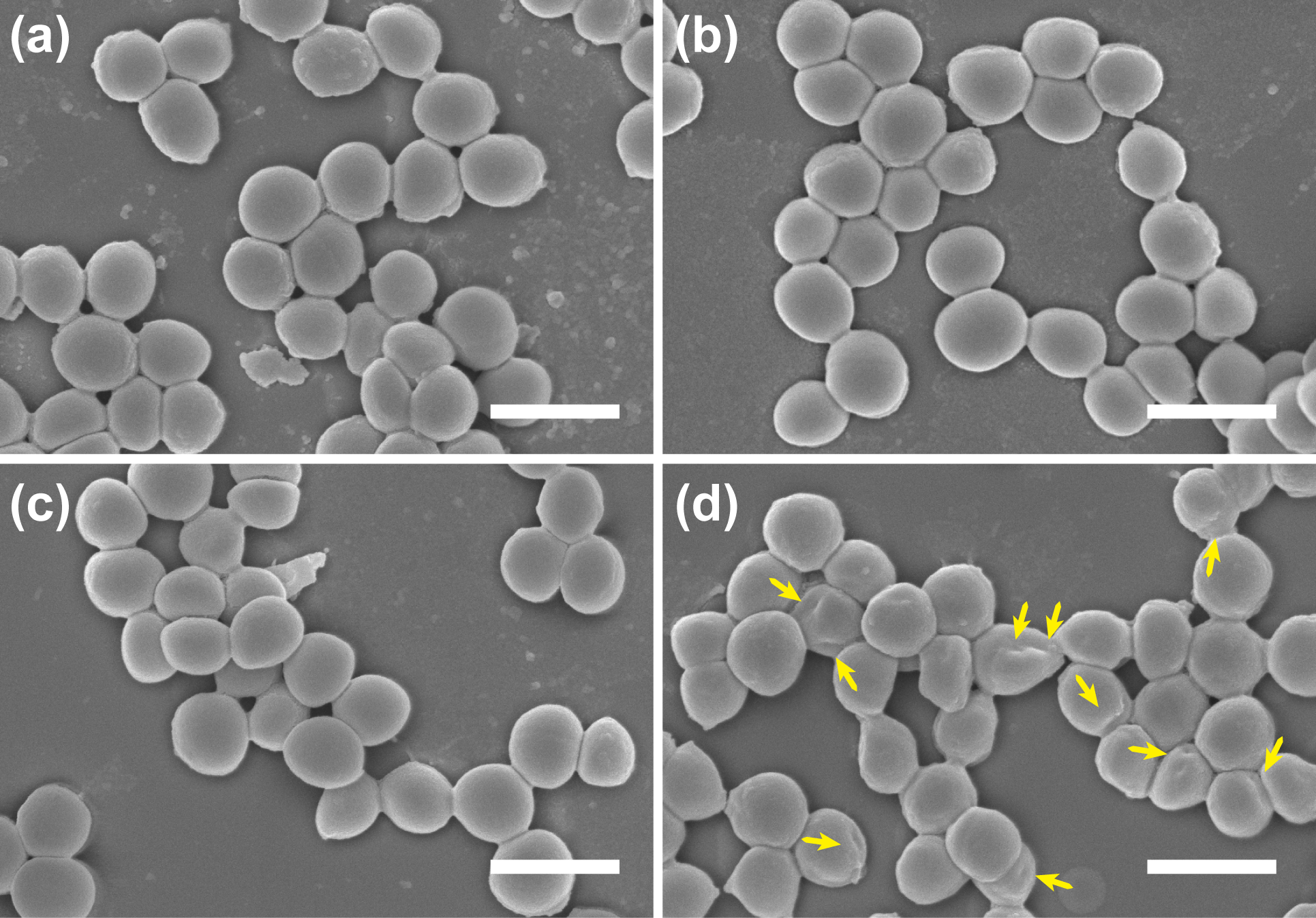

Supplement: S5 Fig — (a) Control, (b) 16 μM vancomycin, (c) 64 μM vancomycin and (d) 64 μM COE-D8. Scale bars correspond to 1 μm. (TIF) [file pone.0224816.s005.tif]
